# Dr. Syam Babu

**Published:** 2010

**Authors:** Mohan Bhaskar, K Jhansi Laksmi Devi

**Affiliations:** Guntur

**Figure:**
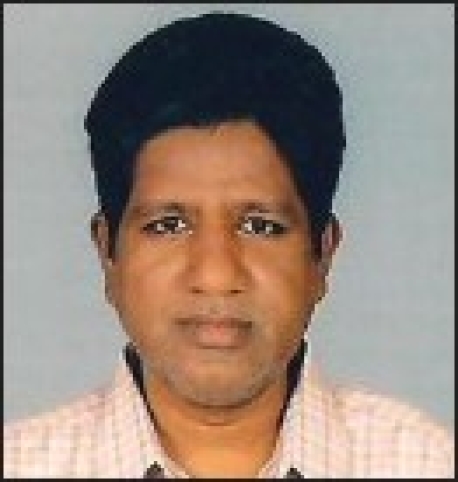


Dr. Syam Babu was born on 2^nd^ April 1967 at Nuzwid in Krishna District of Andhra Pradesh. He completed his schooling from Tadikonda, in Guntur district. He completed his MBBS from Guntur Medical College, Guntur in 1990 and later did his DA from the same institute. Dr. Syam Babu was Life member of the ISA, Guntur branch. He left for his heavenly abode on 29^th^ May 2010. At that time, he was working as a consultant anaesthesiologist at the Amaravathi Institute of Medical Sciences, Guntur.

Dr. Syam Babu is succeeded by his wife, Mrs. P. Swarna Latha, who is working as a junior lecturer and two children, N. Sunder Emmanuel and N. Sarah Supriya.

May his soul rest in peace.

